# Sensitive and long-term monitoring of intracellular microRNAs using a non-integrating cytoplasmic RNA vector

**DOI:** 10.1038/s41598-017-12847-w

**Published:** 2017-10-04

**Authors:** Masayuki Sano, Manami Ohtaka, Minoru Iijima, Asako Nakasu, Yoshio Kato, Mahito Nakanishi

**Affiliations:** 1 Biotechnology Research Institute for Drug Discovery, National Institute of Advanced Industrial Science and Technology (AIST), Central 5, 1-1-1 Higashi, Tsukuba, Ibaraki, 305-8565 Japan; 2 Biomedical Research Institute, National Institute of Advanced Industrial Science and Technology (AIST), Central 6, 1-1-1 Higashi, Tsukuba, Ibaraki, 305-8566 Japan

## Abstract

MicroRNAs (miRNAs) are small noncoding RNAs that modulate gene expression at the post-transcriptional level. Different types of cells express unique sets of miRNAs that can be exploited as potential molecular markers to identify specific cell types. Among the variety of miRNA detection methods, a fluorescence-based imaging system that utilises a fluorescent-reporter gene regulated by a target miRNA offers a major advantage for long-term tracking of the miRNA in living cells. In this study, we developed a novel fluorescence-based miRNA-monitoring system using a non-integrating cytoplasmic RNA vector based on a replication-defective and persistent Sendai virus (SeVdp). Because SeVdp vectors robustly and stably express transgenes, this system enabled sensitive monitoring of miRNAs by fluorescence microscopy. By applying this system for cellular reprogramming, we found that miR-124, but not miR-9, was significantly upregulated during direct neuronal conversion. Additionally, we were able to isolate integration-free human induced pluripotent stem cells by long-term tracking of let-7 expression. Notably, this system was easily expandable to allow detection of multiple miRNAs separately and simultaneously. Our findings provide insight into a powerful tool for evaluating miRNA expression during the cellular reprogramming process and for isolating reprogrammed cells potentially useful for medical applications.

## Introduction

MicroRNAs (miRNAs) are a class of small noncoding RNAs that act as post-transcriptional regulators of gene expression^1^. Many miRNAs are evolutionarily conserved among different organisms and play critical roles in controlling various biological processes, including metabolism, proliferation, and differentiation^2,3^. Comprehensive expression analyses show that different types of cells express unique sets of miRNAs^4,5^. Importantly, some miRNAs are highly abundant in specific cell types and in many cases are directly involved in determining cell identity^3,5^.

Because of their unique expression profiles, miRNAs have been suggested to serve as potential molecular markers in stem cell research^6–8^. Stem cells have the ability to self-renew and differentiate into various types of committed tissue-specific cells. Thus far, numerous protocols for the *in vitro* differentiation of stem cells have been explored to develop potential cell sources for clinical applications, including transplantation therapy^9^. Recently, cellular reprogramming, through which forced expression of defined factors induces cell-fate conversion, has been extensively studied to obtain human induced pluripotent stem cells (hiPSCs) or desired tissue-specific cells^10,11^. However, currently available protocols often produce cell mixtures exhibiting various stages of differentiation. Therefore, specific molecular markers are commonly used to isolate target cells from highly heterogeneous cell populations. Although cell-specific proteins are widely used in this context, recent studies demonstrated that miRNAs can also serve as unambiguous molecular markers^12^. miR-302a, miR-122, and miR-208a can be used to specifically identify embryonic stem cells (ESCs), hepatocytes, and cardiomyocytes, respectively^5,8,13^, and, interestingly, miR-375 can be used as a marker for isolation of insulin-producing cells lacking available specific surface markers^8^.

With the identification of miRNAs as novel molecular markers, a sophisticated system for detecting intracellular miRNAs is in high demand. To examine miRNA expression, northern blots, microarrays, and reverse-transcription PCR (RT-PCR) are broadly exploited as standard techniques^14^, and *in situ* hybridisation and molecular beacons can visualise miRNA expression in cultured cells or *in vivo* ^14^. However, the transient nature of these approaches is unlikely to be suitable for tracking miRNAs over time during differentiation or cellular reprogramming processes. By contrast, a fluorescence-based imaging system containing a fluorescent reporter gene harbouring multiple binding sites for an miRNA of interest in its 3′ untranslated region (UTR) offers a major advantage for tracking of miRNAs in living cells^13,14^. Because binding of miRNA to its target sequences results in inhibition of reporter synthesis, miRNA expression can be readily evaluated based on the decrease in fluorescence intensity. Furthermore, systems based on chromosomal-integrative vectors can stably express reporter genes, thereby allowing long-term monitoring of miRNAs^13,15,16^. However, the activities of cellular and viral promoters, which are commonly used to drive reporter gene expression, vary considerably depending on cell type, genomic context, and epigenetic control^17–20^. Such characteristics might impede sensitive and stable monitoring of miRNAs during cell differentiation and reprogramming processes. Additionally, although these systems have been successfully applied for isolation of hiPSCs after somatic cell reprogramming^21,22^, chromosomal integration of reporter genes is a critical disadvantage for the safe use of hiPSC-derived cells for clinical applications.

To overcome these limitations, we designed a novel fluorescence-based miRNA-monitoring system using a replication-defective and persistent Sendai virus (SeVdp) vector. SeVdp vectors accommodate multiple transgenes into a single vector backbone and simultaneously deliver these genes into target cells^23^. In contrast to typical cellular and viral promoters, transgene expression mediated by SeVdp vectors depends entirely upon the activity of a Sendai virus (SeV) RNA-dependent RNA polymerase (RdRp)^24^. Therefore, this vector confers robust and stable expression of transgenes in various types of mammalian cells^23,25,26^. Importantly, SeVdp vectors also enable prolonged transgene expression without chromosomal integration because RdRp persistently replicates the SeVdp RNA genome in the cytoplasm of infected cells^23,25^. Additionally, if needed, the SeVdp genome can be completely erased from infected cells by inhibiting RdRp function, resulting in the ability to obtain transgene-free cells^23^. These unique properties make the SeVdp vector a versatile gene delivery tool for various applications^27^.

In this study, we demonstrated that a novel SeV-based fluorescence-imaging system, termed SeVdp-miR-Sensor, could be used to reliably evaluate miRNA expression in human stem cells and somatic cells. Based on its stable reporter gene expression, SeVdp-miR-Sensor enabled sensitive monitoring of miR-124 and let-7 during direct neuronal conversion and hiPSC generation, respectively. We were able to isolate reprogrammed hiPSCs by tracking let-7 expression, and the subsequent erasure of the SeVdp genome facilitated generation of transgene-free hiPSCs. Furthermore, we showed that SeVdp-miR-Sensor can be easily expanded to detect two distinct miRNAs separately and simultaneously. Our findings offer insight into a powerful tool for evaluating miRNA expression over time during cellular reprogramming and for isolating transgene-free reprogrammed cells.

## Results

### Stable transgene expression mediated by an SeVdp vector during hiPSC differentiation

To design an ideal fluorescence-based imaging system, we considered an optimal vector platform that continuously expresses a reporter gene regardless of cell type. Because the SeVdp vector is capable of stably expressing transgenes in various mammalian cells, we initially ascertained whether the SeVdp vector could maintain robust gene expression during *in vitro* differentiation of hiPSCs. To this end, we prepared a vector encoding Kusabira-Orange (KO) and blasticidin S deaminase (Bs^r^), termed SeVdp(*Bs* ^*r*^ */KO*) (Supplementary Fig. S1a), and hiPSCs were infected with the vector and treated with blasticidin S to obtain cells harbouring the SeVdp(*Bs* ^*r*^ */KO*) genome. We detected KO expression at 54 days post-infection without significant loss of fluorescence intensity (Supplementary Fig. S1b). Importantly, SeVdp(*Bs* ^*r*^ */KO*) infection did not affect the morphological features or proliferative capacity of hiPSCs during long-term culture (Supplementary Fig. S1b). The hiPSCs harbouring the SeVdp(*Bs* ^*r*^ */KO*) were then cultured on a non-adherent plate to induce spontaneous differentiation. Notably, we observed the maintenance of robust KO expression in embryoid bodies (EBs) and further differentiated cells (Supplementary Fig. S1c,d). These results verified the establishment of stable SeVdp-mediated transgene expression in hiPSCs and their differentiated derivatives. This property offers great advantages for long-term tracking of reporter gene expression during changes in cell state.

### Construction of a fluorescence-based miRNA monitoring system using the SeVdp vector

**Figure 1:**
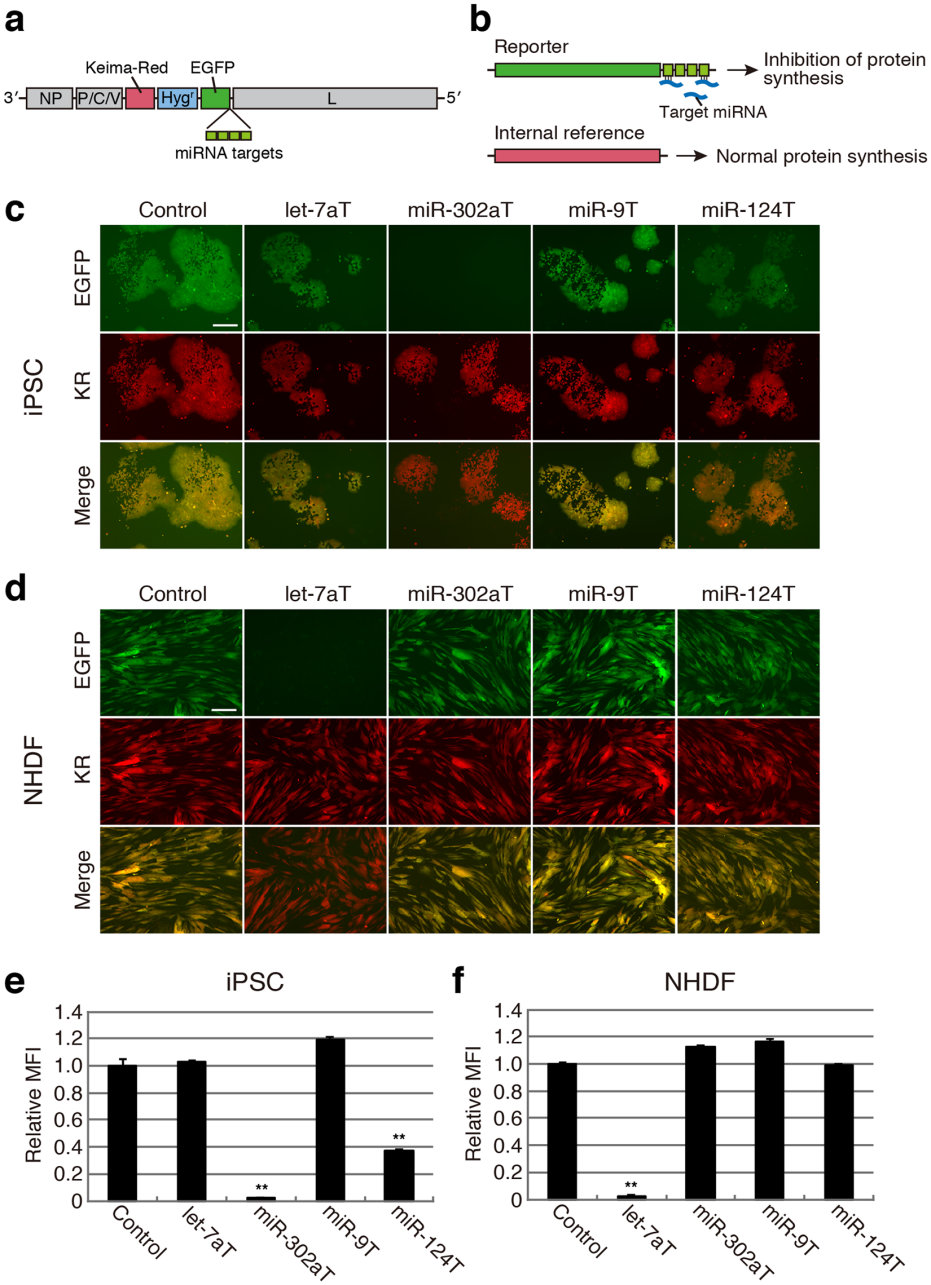
Detection of intracellular miRNAs using SeVdp-miR-Sensor. (**a**) Structure of SeVdp-miR-Sensor. The SeVdp genome encodes Keima-Red (KR), hygromycin B phosphotransferase (Hyg^r^), and EGFP. Four copies of the complementary sequence for the target miRNA were incorporated into the 3′ UTR of the *EGFP* gene. (**b**) Dual-fluorescence-based imaging to evaluate miRNA expression. *EGFP* and *KR* were used as reporter and internal reference genes, respectively. (**c**) Evaluation of miRNA expression in hiPSCs. EGFP and KR expression was analysed by fluorescence microscopy at 6 days post-infection with SeVdp-miR-Sensor. Images with pseudo-colour [green (EGFP), red (KR), and merged] are shown. Control: SeVdp-FlucT; let-7aT: SeVdp-let-7aT; miR-302aT: SeVdp-302aT; miR-9T: SeVdp-9T; and miR-124T: SeVdp-124T. Scale bar: 200 μm. (**d**) Evaluation of miRNA expression in NHDFs. Fluorescent protein expression was analysed at 4 days post-infection, and fluorescence was analysed as described for (**c**). (**e**) Quantitative comparison of miRNA expression in hiPSCs. EGFP levels of KR( + ) cells were analysed by flow cytometry at 6 days post-infection. The EGFP level of cells infected with SeVdp-FlucT was set to 1.0, and relative mean fluorescence intensity (MFI) was evaluated. Data are presented as the mean ± standard deviation of three independent experiments. Statistical analyses were performed using Student’s *t*-test. **P < 0.001 versus Control. (**f**) Quantitative comparison of miRNA expression in NHDFs. The EGFP level of KR( + ) cells was analysed by flow cytometry at 4 days post-infection. Values are the same as those described for (**e**).

To design SeVdp-miR-Sensor, we prepared an SeVdp vector containing a gene encoding enhanced green fluorescent protein (EGFP) and four copies of the complementary sequence of a target miRNA at the 3′ UTR of *EGFP* (Fig. 1a). Binding of the target miRNA to *EGFP* mRNA causes suppression of EGFP synthesis, thereby enabling evaluation of target miRNA expression through measurement of EGFP levels (Fig. 1b). The vector also contained genes encoding Keima-Red (KR) and hygromycin B phosphotransferase (Hyg^r^), enabling KR to be used as an internal reference to ensure reliable interpretation of EGFP levels in infected cells, whereas Hyg^r^ enabled selection of infected cells.

To investigate the potency of SeVdp-miR-Sensor, we constructed vectors containing target sequences for let-7a (SeVdp-let-7aT), miR-302a (SeVdp-302aT), miR-9 (SeVdp-9T), or miR-124 (SeVdp-124T), as well as a vector containing complementary sequences for a portion of the firefly luciferase gene (SeVdp-FlucT) as a control. Initially, we infected hiPSCs with each sensor vector and examined EGFP expression by fluorescence microscopy. We observed decreases in the EGFP signal in SeVdp-302aT-infected hiPSCs, whereas that of SeVdp-let-7aT- and SeVdp-9T-infected hiPSCs was comparable to that of SeVdp-FlucT-infected hiPSCs (Fig. 1c). Additionally, the EGFP signal of SeVdp-124T-infected hiPSCs was also decreased, but the extent of the reduction was less than that observed in SeVdp-302aT-infected hiPSCs. Quantitative RT-PCR (qRT-PCR) analysis indicated that both miR-302a and miR-124 were expressed in hiPSCs (Supplementary Fig. S2a,b). Particularly high levels of miR-302a expression were detected in hiPSCs as compared with levels observed in normal human dermal fibroblasts (NHDFs), H9-derived neural stem cells (H9-NSCs), and Wharton’s jelly stem cells (WJSCs). We observed robust KR expression in all infected cells, indicating that KR was capable of use as a reliable internal reference (Fig. 1c). We also infected NHDFs, H9-NSCs, and WJSCs with each sensor vector, and observed significant decreases in the EGFP signal in SeVdp-let-7aT-infected NHDFs and WJSCs (Fig. 1d and Supplementary Fig. S3a). H9-NSCs exhibited moderately reduced EGFP signals following SeVdp-let-7aT, SeVdp-9T, or SeVdp-124T infection (Supplementary Fig. S3b). Subsequent qRT-PCR analysis indicated that expression levels of let-7a in NHDFs and WJSCs were considerably higher than those in hiPSCs and H9-NSCs, but H9-NSCs had relatively high levels of miR-9 and miR-124 compared to other cells (Supplementary Fig. S2b,c), suggesting that the extent of the reduction in EGFP synthesis should be affected by levels of target miRNAs in the infected cells.

To quantitatively compare EGFP levels between cell types, fluorescent protein expression was measured by flow cytometry. We previously demonstrated that SeVdp vectors could express multiple transgenes at a pre-fixed balance when the transgenes were incorporated onto the same vector backbone^23^. Therefore, we expected that EGFP expression would be reliably normalised along with KR expression. Infected cells exhibiting robust KR intensity were gated, and EGFP levels in those cells were analysed. As shown in Fig. 1e, EGFP levels in SeVdp-302aT- and SeVdp-124T-infected hiPSCs were approximately 40- and 2.7-fold lower than those in SeVdp-FlucT-infected hiPSCs, respectively. Additionally, we observed a ~35-fold reduction in EGFP levels in SeVdp-let-7aT-infected NHDFs as compared with those in SeVdp-FlucT-infected NHDFs (Fig. 1f). Notably, the relative EGFP levels in all cells infected with SeVdp-miR-Sensor correlated well with images obtained by fluorescence microscopy (Fig. 1e,f; Supplementary Fig. S3c,d). We determined a correlation between relative miRNA levels and EGFP suppression, and found that a significant reduction in EGFP signals requires a relatively high level of miRNA expression (Supplementary Fig. S4). These data indicate that SeVdp-miR-Sensor enabled the reliable evaluation of miRNA expression based on EGFP intensity determined by fluorescence microscopy, as well as flow cytometry.

### Monitoring of miR-124 during direct neuronal conversion

We then examined whether SeVdp-miR-Sensor could allow the monitoring of miRNA expression during direct neuronal conversion of mouse embryonic fibroblasts (MEFs). Previous studies demonstrated that ectopic expression of Ascl1, Brn2, and Myt1L efficiently reprogrammed MEFs into induced neuronal cells^28^. Furthermore, the combination of NeuroD1 and these three factors facilitates neuronal conversion of human fibroblasts^29^.

**Figure 2:**
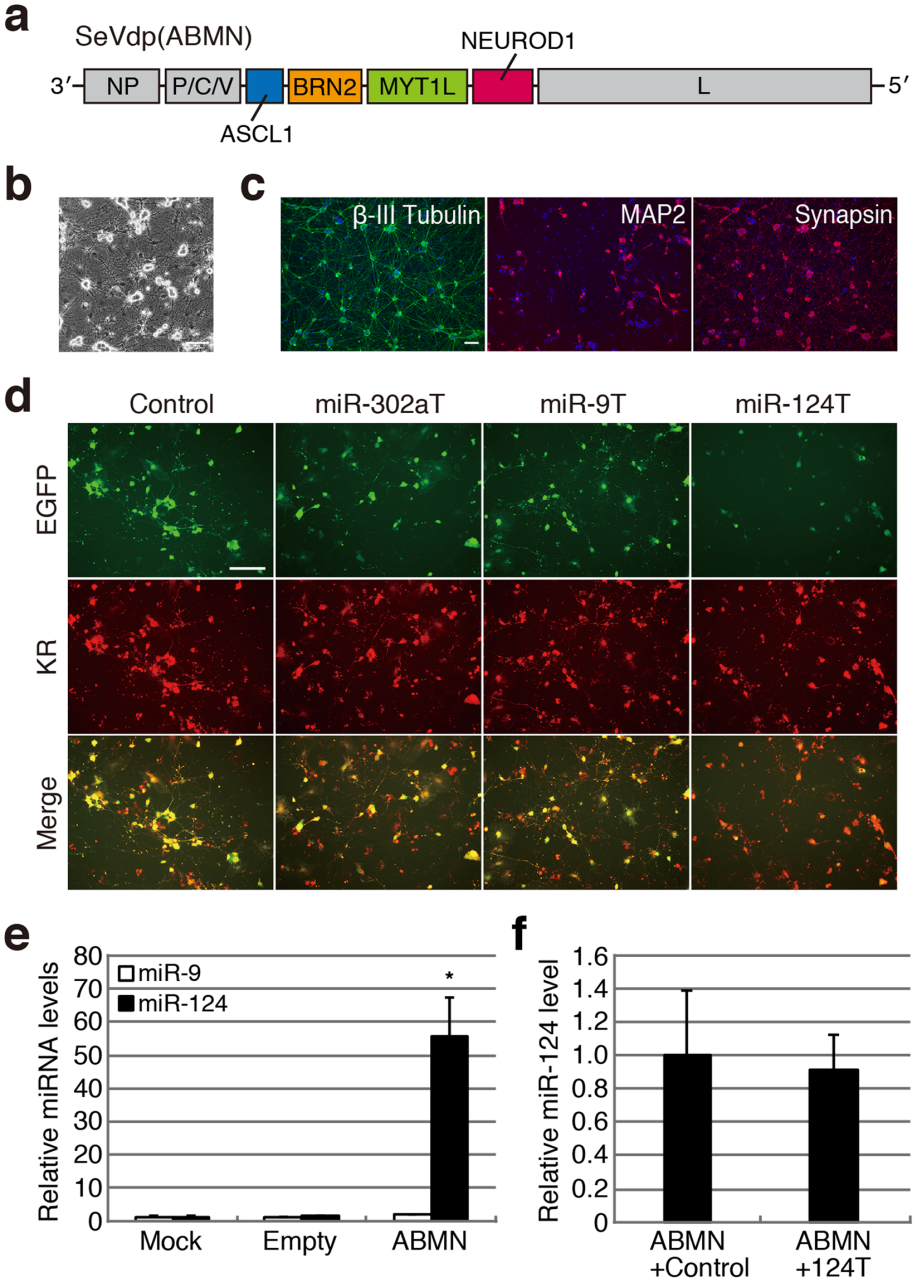
Monitoring of miRNA expression during direct neuronal conversion. (**a**) Structure of the SeVdp(ABMN) vector. Human codon-optimised *ASCL1*, *BRN2*, *MYT1L*, and *NEUROD1* were inserted into the SeVdp vector backbone. (**b**) Efficient neuronal conversion of MEFs using SeVdp(ABMN). A phase-contrast image at 12 days post-infection is shown. Scale bar: 100 μm. (**c**) Neuronal marker expression in converted cells. Expression of neuronal markers was examined at 12 days post-infection. Scale bar: 100 μm. (**d**) Evaluation of miRNA expression during direct neuronal conversion. MEFs were co-infected with SeVdp(ABMN) and SeVdp-miR-Sensor, and fluorescent protein expression was examined by fluorescence microscopy at 8 days post-infection. Images with pseudo-colour are shown. Scale bar: 100 μm. (**e**) Rapid induction of miR-124 expression during neuronal conversion. Expression levels of miR-9 and miR-124 were examined by qRT-PCR at 7 days post-infection of MEFs with SeVdp(ABMN). The miRNA levels of non-infected cells (Mock) were set to 1.0, and relative miRNA levels of the cells infected with SeVdp(ABMN) are indicated. An SeVdp vector containing no transgene was used as a control (Empty). Data are presented as the mean ± standard deviation of three independent experiments. *P < 0.05 versus Mock. (**f**) SeVdp-miR-Sensor does not disturb miRNA expression. MEFs were co-infected with SeVdp(ABMN) and SeVdp-FlucT (ABMN + Control) or SeVdp-124T (ABMN + 124 T), and miR-124 levels were examined by qRT-PCR on day 7. Values are the same as those described for (**e**).

To induce neuronal conversion, we constructed an SeVdp vector containing *ASCL1*, *BRN2*, *MYT1L*, and *NEUROD1* [SeVdp(ABMN)] on the vector backbone (Fig. 2a). Our results indicate that SeVdp(ABMN) infection reprogrammed MEFs into morphologically neuron-like cells, and that these cells expressed typical neuronal markers, including β-III Tubulin, MAP2, and Synapsin I (Fig. 2b,c). Additionally, calcium imaging analysis indicated rapid calcium dynamics in converted cells (Supplementary Video S1), suggesting that SeVdp(ABMN) efficiently induced direct conversion of MEFs into neuronal cells.

To monitor miRNA expression, MEFs were co-infected with SeVdp(ABMN) and SeVdp-miR-Sensor, including SeVdp-FlucT, SeVdp-302aT, SeVdp-9T, and SeVdp-124T, and fluorescent protein expression was examined at 8 days post-infection. We observed a significant reduction in EGFP signal in SeVdp-124T-infected MEFs, whereas SeVdp-302aT- and SeVdp-9T-infected MEFs exhibited EGFP signals similar to those observed in SeVdp-FlucT-infected MEFs (Fig. 2d). Time-lapse imaging revealed reduced EGFP expression in outgrowing neurites at 5 days after co-infection with SeVdp-124T and SeVdp(ABMN) (Supplementary Video S2a,b). Additionally, EGFP expression further decreased and became difficult to detect on day 7. By contrast, we observed EGFP expression in outgrowing neurites from MEFs infected with SeVdp-scrT, which contained four copies of the scrambled sequence in the 3′ UTR of *EGFP* (Supplementary Video S2c,d). These results suggest that miR-124 expression was rapidly upregulated during SeVdp(ABMN)-mediated MEF conversion and that miR-124 continued to accumulate in neuronal cells over time. Notably, qRT-PCR analysis indicated that miR-124 levels were significantly upregulated at 7 days post-SeVdp(ABMN) infection, whereas miR-9 levels remained comparable to levels observed in uninfected MEFs (Fig. 2e). Furthermore, we found that SeVdp-124T infection did not alter overall levels of miR-124 in the converted cells, suggesting that SeVdp-miR-Sensor did not appreciably disturb target miRNA expression (Fig. 2f). These data indicate that SeVdp-miR-Sensor effectively allowed the tracking of miRNA expression during direct neuronal conversion.

### Monitoring of let-7 during iPSC generation and isolation of transgene-free hiPSCs

Ectopic expression of Oct4, Sox2, Klf4, and c-Myc can reprogram human somatic cells into hiPSCs; however, this reprogramming process is relatively inefficient. Previously, a lentiviral vector containing a *GFP* gene connected with the target sequences of let-7a at the *GFP* 3′ UTR was exploited to isolate hiPSCs following somatic cell reprogramming^22^. Because let-7 family members are highly expressed in differentiated cells, but not in hiPSCs^30^, only reprogrammed hiPSCs harbouring the vector exhibit GFP fluorescence. Although this system definitively distinguished hiPSCs from partially reprogrammed cells through examination of GFP expression, chromosomal integration of the reporter gene by the lentiviral vector is unlikely to be suitable for preparing cells derived from the hiPSCs for future medical applications.

**Figure 3:**
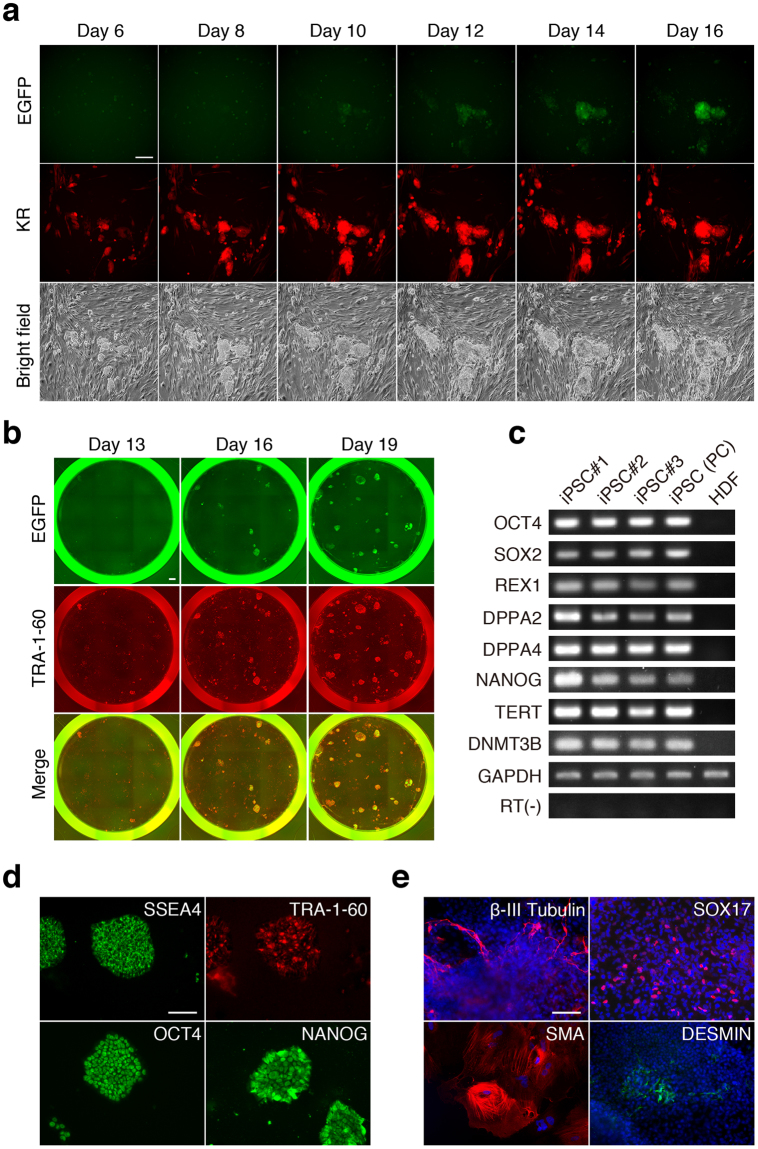
Monitoring of let-7 expression during hiPSC generation. (**a**) Time-lapse analysis of let-7 expression during hiPSC generation. NHDFs were co-infected with SeVdp(KOSM) and SeVdp-let-7aT, and fluorescent protein expression was monitored on the indicated days. Scale bar: 200 μm. (**b**) Comparison of TRA-1-60 and let-7 expression during hiPSC generation. NHDFs were co-infected with SeVdp(KOSM) and SeVdp-let-7aT(KR-), and EGFP and TRA-1-60 expression was examined on days 13, 16, and 19. Scale bar: 900 μm. (**c**) ESC-marker expression in hiPSCs. The expression of ESC- marker genes in three hiPSC clones (#1–3) was analysed by RT-PCR. As positive and negative controls, ESC-marker gene expression in established hiPSCs [iPSC (PC)] and NHDF (HDF) was examined, respectively. RT(-) indicates minus-RT control using the *GAPDH* primer set for PCR. Full-length gel images are presented in Supplementary Information. (**d**) Expression of ESC-marker proteins in hiPSCs. The expression of SSEA4, TRA-1-60, OCT4, and NANOG in an hiPSC clone (#2) was analysed by immunofluorescence staining. Scale bar: 200 μm. (**e**) *In vitro* differentiation of hiPSCs. The hiPSC clone (#1) differentiated into three germ layers: ectoderm (β-III Tubulin), endoderm (SOX17), and mesoderm [smooth muscle actin (SMA) and DESMIN]. Scale bar: 100 μm.

To overcome this limitation, we attempted the same approach using SeVdp-miR-Sensor. We previously demonstrated that the SeVdp vector containing *KLF4*, *OCT4*, *SOX2*, and *c-MYC* [SeVdp(KOSM)] efficiently reprogrammed human somatic cells into hiPSCs^31,32^. To examine whether SeVdp-miR-Sensor could monitor let-7 expression during hiPSC generation, NHDFs were co-infected with SeVdp(KOSM) and SeVdp-let-7aT, and the cells were cultured on feeder cells. Time-lapse imaging revealed that the EGFP signal of SeVdp-let-7aT-infected NHDFs was detectable at ~10 days post-infection, and that the intensity continued to increase gradually (Fig. 3a), suggesting a significant reduction in let-7 expression over the course of reprogramming. By contrast, SeVdp-FlucT-infected cells continuously expressed EGFP and KR, even in reprogrammed colonies (Supplementary Fig. S5a). These data indicate that SeVdp-let-7aT enabled long-term monitoring of let-7 expression during hiPSC generation.

As a reliable pluripotency marker, TRA-1-60 is routinely used to identify fully reprogrammed hiPSCs^33^. To compare the kinetics of TRA-1-60 and let-7 expression, we prepared an SeVdp-let-7aT(KR−) variant lacking the KR reporter from the SeVdp-let-7aT vector (Supplementary Fig. S5b), followed by co-infection of NHDFs with SeVdp(KOSM) and SeVdp-let-7aT(KR−) and culturing under feeder-free conditions. Examination of TRA-1-60 expression by immunofluorescence staining at 13, 16, and 19 days post-infection revealed that TRA-1-60 was expressed on day 13, although colonies did not appreciably express EGFP (Fig. 3b); however, EGFP( + ) colonies were detected on day 16, with the number of these colonies increasing by day 19. Interestingly, EGFP expression was restricted to large, expanding colonies, whereas TRA-1-60 expression was also detectable in small colonies (Fig. 3b and Supplementary Fig. S5c), suggesting that let-7 would be a more suitable marker for identifying hiPSCs during relatively late stages of reprogramming as compared with TRA-1-60.

Previously, we showed that blocking the replication of the SeVdp genome using short interfering RNA (siRNA) against the SeV polymerase gene facilitated erasure of the SeVdp genome from iPSC colonies, resulting in generation of transgene-free iPSCs^23^. Therefore, we selected EGFP( + ) colonies after infection of NHDFs with SeVdp(KOSM) and SeVdp-let-7aT and transfected these cells with siRNA against the SeV polymerase *P* gene (siP234) to remove the SeVdp genomes from the colonies. Following repeated treatment with siP234, we confirmed that expression of SeV nucleocapsid protein (*NP*) mRNA was not detected by qRT-PCR, indicating that the SeVdp genomes were effectively erased from the hiPSC clones (Supplementary Fig. S6a). Additionally, we observed that the clones expressed typical ESC marker genes (Fig. 3c,d; Supplementary Fig. S6b), and that they exhibited pluripotency to differentiate into all three germ layers *in vitro* (Fig. 3e and Supplementary Fig. S6c). These data suggest that SeVdp-miR-Sensor facilitated the isolation of reprogrammed hiPSCs, and that subsequent elimination of SeVdp genomes enabled us to obtain transgene-free hiPSCs.

### Simultaneous detection of two distinct miRNAs using a triple-fluorescence-based SeVdp-miR-Sensor

**Figure 4:**
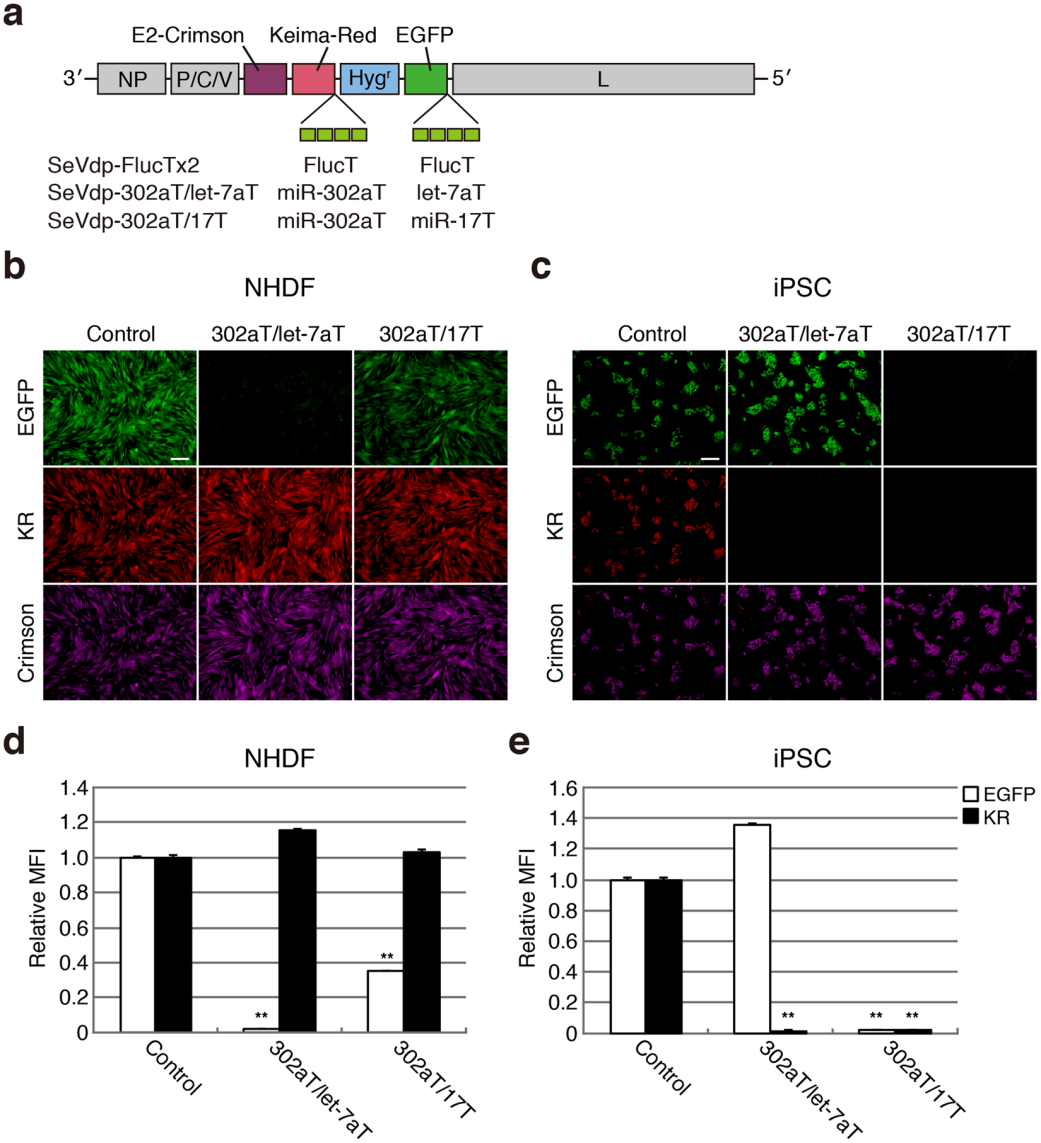
Detection of two distinct miRNAs using triple-fluorescence-based SeVdp-miR-Sensor. (**a**) Structure of the SeVdp genome encoding E2-Crimson, KR, Hyg^r^, and EGFP. Four copies of the complementary sequence for miR-302a, miR-17, or let-7a were incorporated into the 3′ UTRs of the *KR* and *EGFP* genes to construct the SeVdp-302aT/let-7aT and SeVdp-302aT/17T vectors. As a control, the SeVdp-FlucTx2 vector containing four copies of the complementary sequence for a portion of the firefly luciferase gene was used. (**b**) Evaluation of miRNA expression in NHDFs. NHDFs were infected with SeVdp-FlucTx2 (Control), SeVdp-302aT/let-7aT (302aT/let-7aT), or SeVdp-302aT/17T (302aT/17T), and the expression of EGFP, KR, and E2-Crimson (Crimson) was examined by fluorescence microscopy at 2 days post-infection. Images with pseudo-colour [green (EGFP), red (KR), and magenta (Crimson)] are indicated. Scale bars: 200 μm. (**c**) Evaluation of miRNA expression in hiPSCs. hiPSCs were infected with one of the vectors, and fluorescence was analysed as described for (**b**). (**d**) Quantitative comparison of miRNA expression in NHDFs. EGFP and KR expression in E2-Crimson( + ) NHDFs was analysed by flow cytometry at 4 days post-infection. EGFP and KR levels in cells infected with SeVdp-FlucTx2 were set to 1.0, and the relative MFI was evaluated. Data are presented as the mean ± standard deviation of three independent experiments. **P < 0.001 versus Control. (**e**) Quantitative comparison of miRNA expression in hiPSCs. Experimental procedures and values are the same as described for (**d**).

Fluorescence-based imaging systems can potentially enable detection of multiple miRNAs through the use of different sets of fluorescent proteins. However, most gene delivery platforms have a limitation in that they harbour multiple expression cassettes on a single vector backbone. Therefore, we attempted to design a triple-fluorescence-based monitoring system by constructing an SeVdp vector encoding three different fluorescent proteins (EGFP, KR, and E2-Crimson). Subsequent incorporation of four copies of the target sequence for miR-302a, let-7a, or miR-17 into the 3′ UTR of *KR* or *EGFP* created two different sensor vectors, SeVdp-302aT/let-7aT and SeVdp-302aT/17T (Fig. 4a), with SeVdp-302aT/let-7aT capable of being used to simultaneously evaluate miR-302 and let-7 expression, and SeVdp-302aT/17T capable of being used to simultaneously evaluate miR-302 and miR-17 expression. We also prepared SeVdp-FlucTx2, which contained four copies of the complementary sequence for the portion of the firefly luciferase gene at the 3′ UTRs of *KR* and *EGFP*. In all of the vectors, E2-Crimson was used as an internal reference to identify vector-infected cells. NHDFs and hiPSCs were infected with SeVdp-FlucTx2, SeVdp-302aT/let-7aT, or SeVdp-302aT/17T, and fluorescent protein expression was examined by fluorescence microscopy. We detected KR expression in SeVdp-302aT/let-7aT-infected NHDFs, whereas the EGFP signal was significantly diminished (Fig. 4b). By contrast, in hiPSCs, this vector expressed EGFP at normal levels, but exhibited minimal KR expression (Fig. 4c). Additionally, we observed slight reductions in EGFP expression in SeVdp-302aT/17T-infected NHDFs (Fig. 4b), whereas KR and EGFP expression in hiPSCs was strongly suppressed (Fig. 4c). We also observed normal levels of KR and EGFP expression in SeVdp-FlucTx2-infected NHDFs and hiPSCs (Fig. 4b,c). To quantitatively compare the fluorescence intensities, KR and EGFP expression in E2-Crimson( + ) cells was measured by flow cytometry. The relative fluorescence intensities of the reporter proteins correlated well with images obtained by fluorescence microscopy (Fig. 4d,e; compared with Fig. 4b,c). These data indicated that hiPSCs highly expressed miR-302 and miR-17, whereas NHDFs highly expressed let-7 accompanied by modest levels of miR-17 expression. Subsequent qRT-PCR analysis revealed that miR-17 was expressed in both hiPSCs and NHDFs, but its expression level was higher in hiPSCs than in NHDFs (Supplementary Fig. S2d). These results suggest that SeVdp-miR-Sensor was expandable to allow separate and simultaneous evaluation of the expression of multiple miRNAs.

## Discussion

Here, we present a novel fluorescence-based imaging system, SeVdp-miR-Sensor, for monitoring miRNA expression in living cells, based on its ability to inhibit the expression of fluorescence-reporter genes. In this system, we employed two distinct fluorescent proteins, EGFP and KR, as a reporter and internal reference, respectively, enabling us to reliably interpret miRNA expression in individual cells. A similar approach was previously described using various vector platforms, including plasmids, retroviral and lentiviral vectors, and the *piggyBac*-transposon^34–37^. These vectors require cellular and viral promoters to drive the expression of reporter genes. Elongation factor-1α (EF1α) and β-actin promoters exhibit potent transcriptional activities in mouse and human ESCs^17,19,38^, and phosphoglycerate kinase and ubiquitin C (UbC) promoters direct robust expression in murine neocortical cultures^39^. However, the transcriptional activities of these promoters would likely be altered based on cell type and culture conditions. The activities of the EF1α and UbC promoters gradually decreased during the long-term culture of hESCs^19^, with the EF1α promoter losing most of its activity during the neuronal differentiation of mESCs^17^. These characteristics represent clear disadvantages for stable transgene expression during cell conversion processes. In contrast to typical expression systems, SeVdp employs RdRp and *cis*-acting elements incorporated into the SeVdp genome to synthesise mRNAs^24,40^. Given the availability of host-independent transcriptional machinery, SeVdp vectors are able to confer stable transgene expression in a broad range of mammalian cells and even during hiPSC differentiation (Supplementary Fig. S1).

Previous reports suggested that miRNA-mediated target suppression requires a threshold miRNA concentration^13^. Highly expressed miRNAs strongly repress target expression, whereas in some cases less abundant miRNAs have no appreciable suppressive activity^13,41^. Additionally, miRNA family members, such as those of the let-7 family, give rise to robust suppressive activity because all of the family members cooperatively inhibit target-gene expression^41^. Thus, it is likely that the suppression level of the fluorescent protein is not always correlated with actual amounts of a target miRNA in cells. Consistent with previous findings^13,41^, we found that SeVdp-miR-Sensor was able to sensitively respond to a relatively high level of miRNA, and the extent of reduction in fluorescence intensity was considerably affected by the relative levels of the target miRNAs (Supplementary Fig S4). In most cases, physiologically functional miRNAs are relatively abundant, and miRNA family members exhibit similar expression patterns in specific cell types^5,30^. Although we examined the expression of several tissue-specific miRNAs in this study, we expect that SeVdp-miR-Sensor can be widely used for evaluation of the expression of miRNA with relatively high abundance in specific cell states, even during differentiation or cellular reprogramming. Previous reports suggested that the position and number of miRNA target sites incorporated into the reporter gene influences the level of target suppression^8,13^. Thus, future refinement of SeVdp-miR-Sensor may improve responsiveness to even relatively small amounts of miRNAs.

Following discovery of the reprogramming of somatic cells into iPSCs, several lines of evidence demonstrated that ectopic expression of lineage-related factors could directly reprogram fibroblasts into tissue-specific cells^10^. Although previous studies often employed lentiviral vectors to express defined factors, we showed that the SeVdp vector efficiently induced the neuronal conversion of MEFs (Fig. 2b,c). Additionally, we found that simple co-infection with SeVdp(ABMN) and SeVdp-124T enabled monitoring of miR-124 expression during direct neuronal conversion (Fig. 2d). miR-124 is enriched in the animal brain and plays a critical role in the developing central nervous system^42,43^. Because miR-124 is specifically expressed in neurons, but not astrocytes, it can be exploited as a neuron-specific molecular marker. Our time-lapse imaging results using SeVdp-124T revealed a spatiotemporal expression pattern associated with miR-124 during direct neuronal conversion, suggesting that SeVdp-124T might contribute to the efficacious evaluation of various protocols related to neuronal differentiation. In contrast to miR-124, we did not observe miR-9 expression over time during neuronal conversion (Fig. 2d,e), even though miR-9 reportedly exhibits strong expression in the brain and promotes neural differentiation^44^. Interestingly, overexpression of miR-9 and its opposite strand (miR-9*) in combination with miR-124 facilitates the reprogramming of human fibroblasts into neurons^45^, suggesting a positive role for miR-9 in direct neuronal conversion. Although the exact effect of miR-9 expression during neuronal conversion remains elusive, its expression might be affected by induction or culture conditions (e.g., defined factors, medium, or feeder cells). Alternatively, miR-9 might be preferentially expressed in specific neuronal subtypes. It will be interesting to investigate the roles of miR-9 in direct neuronal-conversion processes in future work.

Comprehensive gene expression analyses have revealed that hiPSCs express a unique set of pluripotency genes. Alkaline phosphatase (AP), SSEA4, TRA-1-60, and NANOG have been commonly used as molecular markers to identify hiPSC colonies^12^. Notably, stepwise expression of these marker genes during hiPSC generation facilitates identification of reprogramming states. Although AP and SSEA4 are detected during the early phase of reprogramming, TRA-1-60 and NANOG expression appears during relatively later phases^33^. Here, we observed that significant reductions in let-7 expression occurred later as compared with TRA-1-60 expression during NHDF reprogramming and following infection with SeVdp(KOSM) (Fig. 3b). This result agreed with a previous report by Di Stefano *et al*. showing inverse correlations between let-7 expression and the expression of pluripotency genes, including *LIN28*, *NANOG*, and *REX1*, in reprogrammed colonies^22^. Here, we found that large, expanding colonies exhibited significantly decreased let-7 expression as compared with that observed in small colonies (Fig. 3b). These findings suggest that let-7 constituted an effective molecular marker for identifying fully reprogrammed hiPSCs.

In contrast to chromosomal-integrative vectors, such as lentiviral vectors, SeVdp vectors do not require chromosomal integration of transgenes for stable expression. This enabled us to select reprogrammed colonies using SeVdp-let-7aT and to subsequently obtain transgene-free hiPSCs by simply erasing the SeVdp genome from the colonies. This protocol might allow mature hiPSCs to be easily discerned from partially reprogrammed colonies and might enable the preparation of hiPSCs potentially applicable for medical applications. Recently, a non-integrating episomal vector was exploited to construct a fluorescence-based imaging system to evaluate miRNA activity^46^. Although this system allowed long-term monitoring of miR-302a-5p expression during hiPSC differentiation, episomal vectors may be rapidly cleared from highly proliferating cells^46^. By contrast, the replication of the SeVdp genome is remarkably stable, even in hiPSCs (Supplementary Fig. S1). Therefore, SeVdp-miR-Sensor enables long-term monitoring of miRNA expression without significant loss of fluorescence intensity.

Changes in cell state are accompanied by concomitant upregulation and downregulation of many miRNAs. Because multiple miRNAs are recognised as potential molecular markers in specific cell states^12^, simultaneous evaluation of miRNA expression would likely provide greater precision in cell identification. Because SeVdp vectors are capable of harbouring multiple transgenes on a single vector backbone^23,47^, we were able to modify SeVdp-miR-Sensor to enable the detection of two distinct miRNAs using three different fluorescent proteins (Fig. 4). Importantly, we were able to evaluate the expression of these miRNAs separately and simultaneously. Recent advances in fluorescence technology allowed us to select various combinations of coloured fluorescent proteins. Therefore, we anticipate that SeVdp-miR-Sensor might be capable of further expansion to monitor more than two distinct miRNAs using different sets of fluorescence-reporter genes.

In summary, our findings demonstrate that SeVdp-miR-Sensor enabled stable and long-term monitoring of miRNAs in living cells. This system can be exploited to identify specific miRNA expression during cellular reprogramming and subsequently isolate resultant reprogrammed cells. Importantly, SeVdp-miR-Sensor is capable of sensitively monitoring miRNA expression by visualising reporter proteins under fluorescence microscopy. Significant efforts in miRNA profiling have revealed that miRNA expression is dramatically altered during development, differentiation, and under various pathogenic conditions, including those related to cancer^48^, infectious diseases^49^, and neurological diseases^50^. Therefore, we expect that SeVdp-miR-Sensor will contribute to monitoring miRNAs, evaluating cell states, and isolating target cells across a broad range of biological and medical research.

## Methods

### SeVdp-vector production

SeVdp genomic cDNAs were constructed as described previously^23^. cDNAs encoding KO, EGFP, KR, E2-Crimson, and Bs^r^ were amplified by PCR using phKO1-MN1 (Medical & Biological Laboratories, Nagoya, Japan), pEGFP-1 (Takara Bio, Otsu, Japan), phdKeima-Red-S1 (Medical & Biological Laboratories), pE2-Crimson Vector (Clontech, Mountain View, CA, USA), and pCX4-bsr^51^ as templates, respectively. cDNA encoding Hyg^r^ was synthesised by GeneScript (Piscataway, NJ, USA). These cDNAs were used to construct SeVdp(*Bs* ^*r*^/*KO*), SeVdp(*Hyg* ^*r*^/*EGFP*), SeVdp(*KR*/*Hyg* ^*r*^/*EGFP*), and SeVdp(*Crimson*/*KR*/*Hyg* ^*r*^/*EGFP*). To construct SeVdp-302aT, SeVdp-let-7aT, SeVdp-9T, and SeVdp-124T, four copies of the miRNA target sequence were inserted into the 3′ UTR of the *EGFP* gene in the SeVdp(*KR*/*Hyg* ^*r*^/*EGFP*) vector. To construct SeVdp-let-7aT(KR−), four copies of the let-7a target sequence were inserted into the 3′ UTR of the *EGFP* gene in the SeVdp(*Hyg* ^*r*^/*EGFP*) vector. To construct SeVdp-302aT/let-7aT and SeVdp-302aT/17T, four copies of the miRNA target sequence were inserted into the 3′ UTRs of the *KR* and *EGFP* genes in the SeVdp(*Crimson*/*KR*/*Hyg* ^*r*^/*EGFP*) vector. To construct SeVdp-FlucT and SeVdp-FlucTx2, four copies of the complementary sequence for a portion of the firefly luciferase gene^52^ were inserted into the 3′ UTR(s) of the *KR* and/or *EGFP* genes in the SeVdp(*KR*/*Hyg* ^*r*^/*EGFP*) and SeVdp(*Crimson*/*KR*/*Hyg* ^*r*^/*EGFP*) vectors, respectively. To construct SeVdp-scrT, four copies of the scrambled sequence were inserted into the 3′ UTR of the *EGFP* gene in the SeVdp(*KR*/*Hyg* ^*r*^/*EGFP*) vector. The scrambled sequence was designed using siRNA Wizard v3.1 software (InvivoGen, San Diego, CA, USA). The DNA sequences that included miRNA target sequences are listed in Supplementary Table S1. cDNAs encoding codon-optimised ASCL1, BRN2, MYT1L, and NEUROD1 were synthesised by GeneScript and used to construct the SeVdp(ABMN) vector. Preparation of vector packaging cells and the production of vectors were described previously^23,26^.

### Cell culture and viral infection

NHDFs (KURABO, Osaka, Japan) and MEFs were cultured in Dulbecco’s modified Eagle’s medium (DMEM; Sigma-Aldrich, St. Louis, MO, USA) supplemented with 10% fetal bovine serum (FBS: HyClone Laboratories, Logan, UT, USA) and penicillin-streptomycin (Pen-Strep; Wako, Osaka, Japan). WJSCs (DV biologics, Yorba Linda, CA, USA) were cultured in H-GRO medium (DV biologics). H9-NSCs (Thermo Fisher Scientific, Waltham, MA, USA) were cultured in StemPro NSC SFM (Thermo Fisher Scientific) on a plate coated with CTS CELLstart substrate (Thermo Fisher Scientific). hiPSCs^26^ were cultured in mTeSR1 (STEMCELL Technologies, Vancouver, Canada) on a plate coated with iMatrix-511 (Nippi, Tokyo, Japan).

hiPSCs were infected with SeVdp(*Bs* ^*r*^/*KO*) at a multiplicity of infection (MOI) of four for 2 h at room temperature, followed by incubation for 1 h at 32 °C. The viral medium was replaced with mTeSR1, and 10 µg/mL blasticidin S (Bs) was added at 4 days post-infection. The cells were continuously cultured in the presence of Bs.

hiPSCs or NHDFs were infected with SeVdp-miR-Sensor at a similar infection rate for 2 h at room temperature, followed by incubation for between 2 h and 6 h at 37 °C. H9-NSCs or WJSCs were infected with SeVdp-miR-Sensor at a similar infection rate for 2 h at room temperature, followed by incubation at 37 °C overnight. Triple-fluorescence-based SeVdp-miR-Sensors were used to infect hiPSCs or NHDFs at an MOI of three for 2 h at room temperature, followed by incubation for 2 h at 32 °C. The viral medium was replaced with the culture medium for each cell type.

### Fluorescence microscopy, flow cytometry, and image acquisition

KO, KR, EGFP, and E2-Crimson were detected using a fluorescence microscope (Axio Observer.A1; Zeiss, Oberkochen, Germany) using customised filters. Fluorescence was analysed with iVision-Mac software (Solution Systems, Funabashi, Japan). Co-imaging of fluorescence for EGFP, AlexaFluor488 (Thermo Fisher Scientific), AlexaFluor555 (Thermo Fisher Scientific), and 4′,6-diamidino-2-phenylindole (DAPI) was performed using Axio Observer or BIOREVO BZ-9000 with a BZ-II analyzer (Keyence, Osaka, Japan). Time-lapse fluorescence microscopy was performed using a DMi8 (Leica, Wetzlar, Germany), and data were analysed with LAS X software (Leica). Flow cytometry was performed using a Gallios flow cytometer (Beckman Coulter, Brea, CA, USA), and the mean fluorescence intensity (MFI) was calculated using Kaluza software (Beckman Coulter).

### Cellular reprogramming

For direct neuronal conversion, MEFs were seeded onto an iMatrix-511-coated plate and infected with SeVdp(ABMN) at an MOI of 18. The cells were then co-infected with SeVdp-miR-Sensor at an MOI of two for evaluation of miRNA expression. Viral medium was replaced with neuronal culture medium [DMEM/Ham’s F12 medium (Sigma-Aldrich), N2 supplement with transferrin (Apo) (Wako), NeuroBrew-21 (Miltenyi Biotech, Bergisch Gladbach, Germany), and Pen-Strep]. The medium was changed every 2 to 3 days.

To generate hiPSCs, NHDFs were co-infected with the SeVdp(KOSM) vector and SeVdp-miR-Sensor (SeVdp-FlucT or SeVdp-let-7aT) at an MOI of four each. The infected cells were then seeded onto mitomycin C-treated MEFs (ReproCELL, Yokohama, Japan) and cultured in Primate ES medium (ReproCELL) with 5 ng/mL basic fibroblast growth factor (Wako). For the feeder-free culture, NHDFs were co-infected with the SeVdp(KOSM) vector and SeVdp-let-7aT or SeVdp-let-7aT(KR−) at an MOI of four each. The infected cells were seeded onto an iMatrix-511-coated plate and cultured in Stem Fit AK02N (Ajinomoto, Tokyo, Japan). To remove the SeVdp genome from reprogrammed colonies, cells were transfected with 40 nM siP234^23^ using Lipofectamine RNAi MAX reagent (Thermo Fisher Scientific) at 27 days post-infection. The transfection was performed three additional times every 2 to 4 days. siP234 was synthesised by GeneDesign (Osaka, Japan)

### RNA expression analysis

Total RNA was extracted using the ISOGEN reagent (Nippon Gene, Tokyo, Japan). For miRNA expression analysis, cDNAs were synthesised using the TaqMan miRNA reverse transcription kit (Applied Biosystems, Foster City, CA, USA), and miRNA levels were determined using the TaqMan miRNA assays (Applied Biosystems). Levels of RNU48 (human) or snoRNA202 (mouse) were used to normalise data. For mRNA expression analysis, total RNA was treated with DNase I (Nippon Gene) to digest residual DNA, and cDNAs were synthesised using the SuperScript III first-strand synthesis system (Thermo Fisher Scientific). PCR was performed using GoTaq Green master mix (Promega, Madison, WI, USA) with primer sets described previously^53,54^. *NP* and *GAPDH* mRNA levels were determined by quantitative real-time PCR (up to 45 cycles) using SsoAdvanced Universal SYBR Green Supermix (Bio-Rad, Hercules, CA, USA) and the following primers: *NP* (Fwd: 5′-CATCCAGATCGTTGGGAACT-3′, Rev: 5′-GAGCTGCCATCTTTGTCTCC-3′), and *GAPDH* (Fwd: 5′-CTTTGGTATCGTGGAAGGACTC-3′, Rev: 5′-GTAGAGGCAGGGATGATGTTCT-3′).

### Immunofluorescence staining

Cells were fixed with 3.7% formaldehyde in phosphate-buffered saline (PBS). After permeablisation with 0.1% to 0.2% Triton X-100/PBS, cells were incubated with a primary antibody, followed by staining with a secondary antibody conjugated with AlexaFluor488 (1:500) or AlexaFluor555 (1:500). The following primary antibodies were used in this study: anti-SSEA4 (1:250; Merck Millipore, Darmstadt, Germany), anti-OCT4 (1:400; Abcam, Cambridge, UK), anti-NANOG (1:20; R&D Systems, Minneapolis, MN, USA), anti-TRA-1-60 (1:250; e-Bioscience, Santa Clara, CA, USA), anti-β-III Tubulin (1:1,000; BioLegend, San Diego, CA, USA), anti-SOX17 (1:200; Abcam), anti-smooth muscle actin (1:250; Dako, Santa Clara, CA, USA), anti-Desmin (1:200; Thermo Fisher Scientific), anti-MAP2 (1:500; Santa Cruz, Dallas, TX, USA), and anti-Synapsin I (1:500; Abcam). Nuclei were counterstained with DAPI using VECTASHIELD mounting medium with DAPI (Vector Laboratories, Burlingame, CA, USA).

### Calcium imaging

Cells were labelled with 10 µg/mL Fluo-4AM (Thermo Fisher Scientific) in DMEM/Ham’s F12 medium (minus phenol red) with GlutaMAX (Thermo Fisher Scientific) for 20 min at 37 °C. The labelling medium was replaced with Ringer’s solution^55^, and fluorescence was immediately monitored by time-lapse fluorescence microscopy.

### *In vitro* differentiation assay

hiPSCs were treated with TrypLE Express (Thermo Fisher Scientific) and transferred onto Nunclon Sphera Microplates (Thermo Fisher Scientific) in Primate ES cell culture medium supplemented with 10 µM Y27632 (Wako). Cells were cultured for 3 to 5 days to allow EB formation. EBs were attached to a gelatin-coated plate and cultured in DMEM supplemented with 10% FBS for an additional 10 to 12 days.

## Electronic supplementary material

Supplementary Information

Supplementary Video S1

Supplementary Video S2a

Supplementary Video S2b

Supplementary Video S2c

Supplementary Video S2d
