# Supplementary material for: Sensitive and long-term monitoring of intracellular microRNAs using a non-integrating cytoplasmic RNA vector
Source: Sci Rep. 2017 Oct 4;7:12673. doi: 10.1038/s41598-017-12847-w (PMC5627244; doi:10.1038/s41598-017-12847-w)

**Supplementary Information**

# Sensitive and long-term monitoring of intracellular microRNAs using a non-integrating cytoplasmic RNA vector

Masayuki Sano[1,*], Manami Ohtaka[1], Minoru Iijima[1], Asako Nakasu[1], Yoshio Kato[2], Mahito Nakanishi[1]

[1]Biotechnology Research Institute for Drug Discovery, National Institute of Advanced Industrial Science and Technology (AIST), Central 5, 1-1-1 Higashi, Tsukuba, Ibaraki 305-8565, Japan

[2]Biomedical Research Institute, National Institute of Advanced Industrial Science and Technology (AIST), Central 6, 1-1-1 Higashi, Tsukuba, Ibaraki 305-8566, Japan

[*]Corresponding author. E-mail: m.sano@aist.go.jp

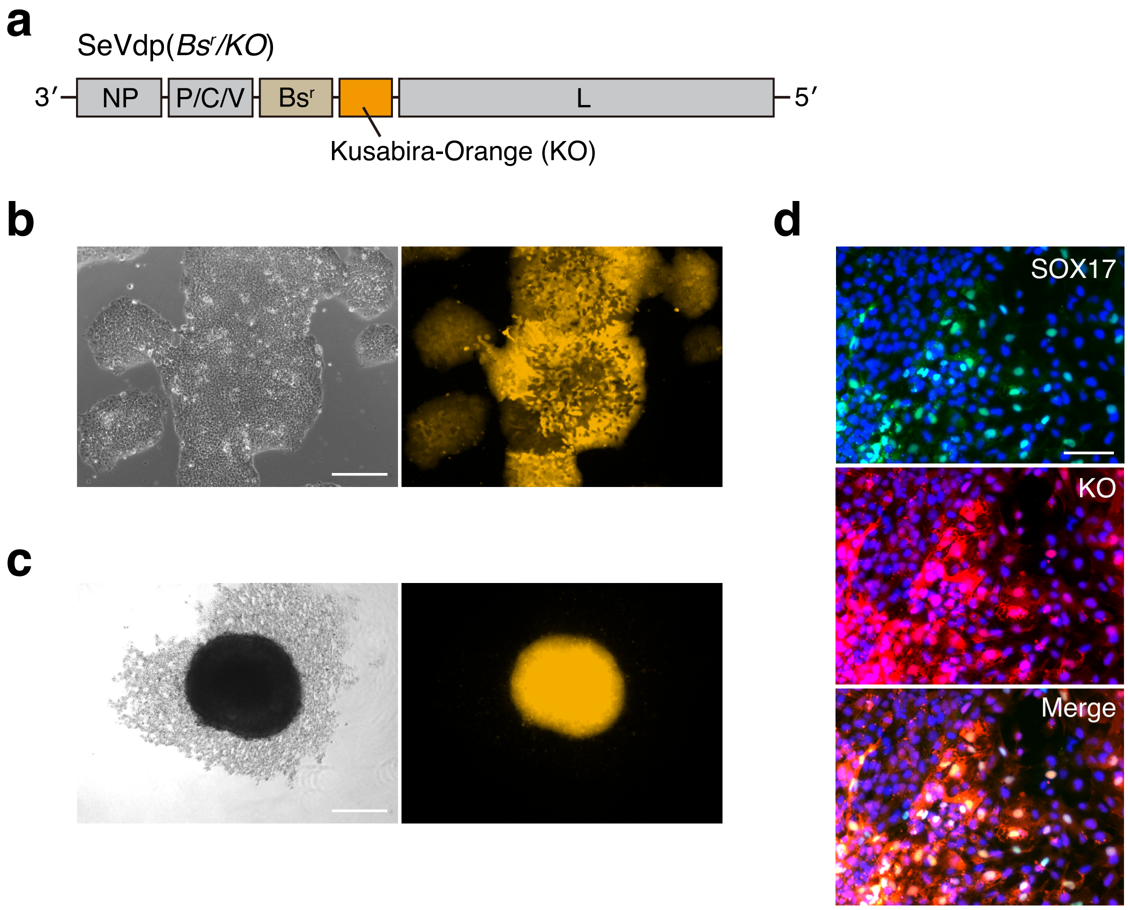


**Supplementary Figure S1. SeVdp-mediated stable transgene expression during hiPSC differentiation.** (a) Structure of the SeVdp(*Bs*$^r$*/KO*) genome encoding blasticidin S deaminase (Bs$^r$) and Kusabira-Orange (KO). The *NP*, *P*, and *L* genes are necessary for the replication of SeVdp RNA genome and transcription. The *P* gene contains multiple open reading frames encoding the P, C, and V proteins. (b) KO expression in hiPSCs. Phase-contrast and fluorescence images are indicated. Scale bar: 200 µm. (c) KO expression in an embryoid body (EB). hiPSCs were cultured on a non-adherent plate, and KO expression was examined after 3 days of culture. Scale bar: 400 µm. (d) KO expression in hiPSC-derived cells. EBs were cultured on a gelatin-coated plate, and KO and SOX17 (endoderm marker) expression was determined. Scale bar: 100 µm.

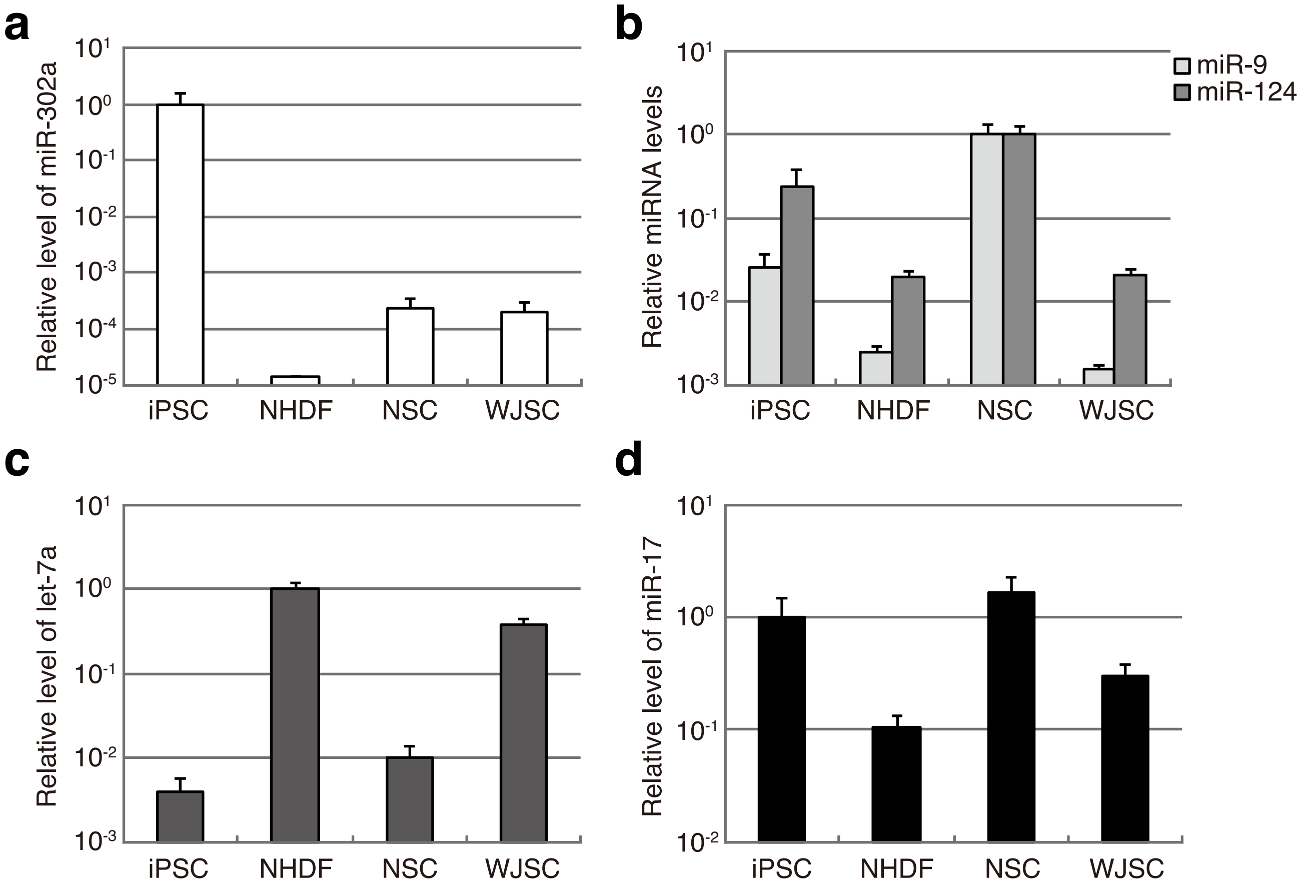


**Supplementary Figure S2. Comparison of miRNA levels in various human cells.** (a) Expression level of miR-302a. Levels of miR-302a in hiPSCs (iPSC), NHDFs (NHDF), H9-NSCs (NSC), and WJSCs (WJSC) were examined by qRT-PCR. The miRNA level in hiPSCs was set to 1.0, and relative miRNA levels are indicated. Data are presented as the mean ± standard deviation of three independent experiments. (b) Expression levels of miR-9 and miR-124. The miRNA levels in H9-NSCs were set to 1.0, and relative miRNA levels are indicated. (c) Expression level of let-7a. The miRNA level in NHDFs was set to 1.0, and relative miRNA levels are indicated. (d) Expression level of miR-17. The miRNA level in hiPSCs was set to 1.0, and relative miRNA levels are indicated. Values are the same as those described for (a).

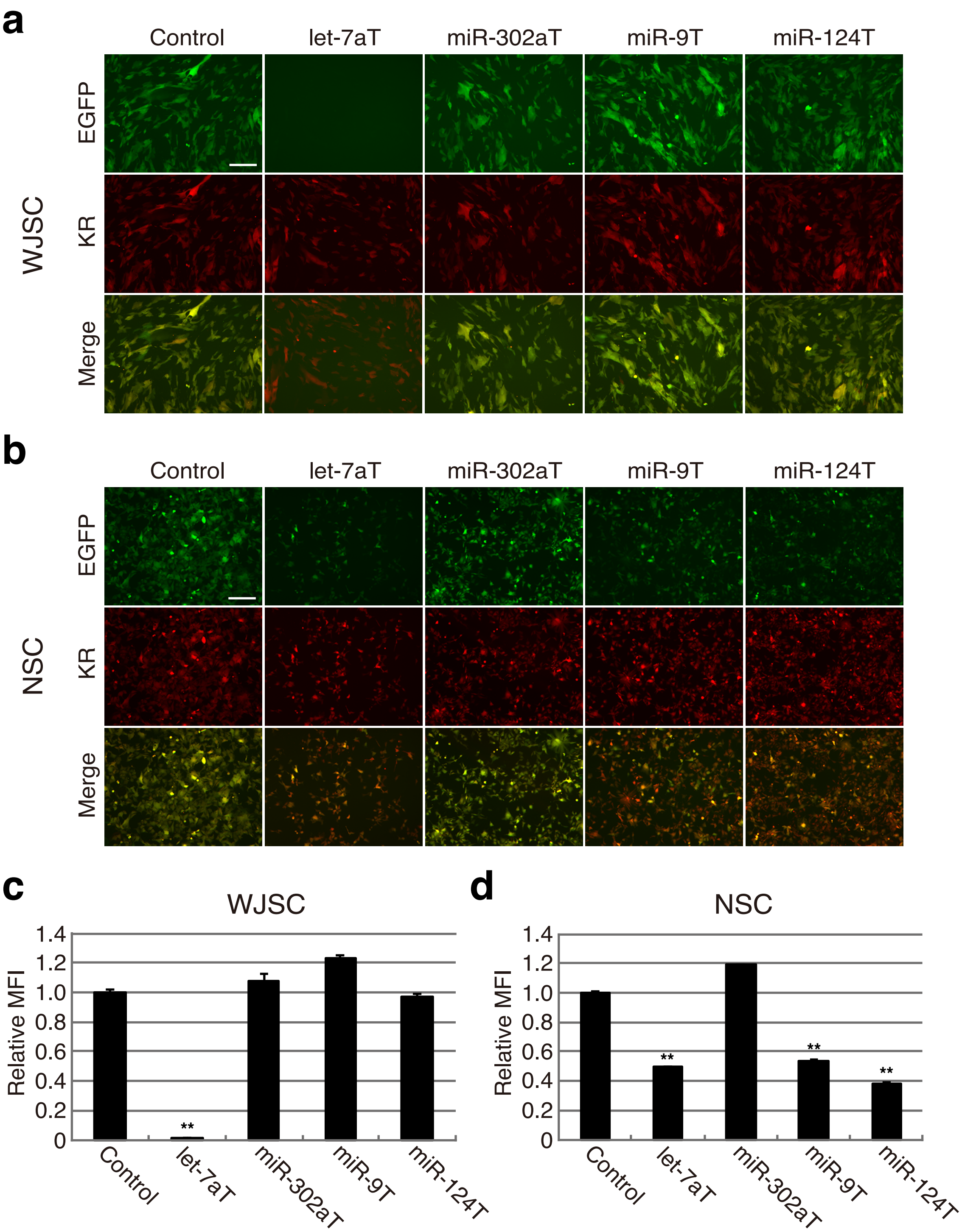


**Supplementary Figure S3. Detection of miRNAs in WJSCs and H9-NSCs using SeVdp-miR-Sensor.** (a) Evaluation of miRNA expression in WJSCs. EGFP and KR expression was analysed at 3 days post-infection with SeVdp-miR-Sensor. Scale bar: 200 μm. (b) Evaluation of miRNA expression in H9-NSCs. The fluorescence was analysed at 3 days post-infection. Scale bar: 200 μm. (c) Quantitative comparison of miRNA expression in WJSCs. EGFP levels of KR(+) cells were analysed by flow cytometry at 3 days post-infection. Data are presented as the mean ± standard deviation of three independent experiments. $^{**}P < 0.001$ versus Control. (d) Quantitative comparison of miRNA expression in H9-NSCs. Experimental procedure and values are the same as described for (c).

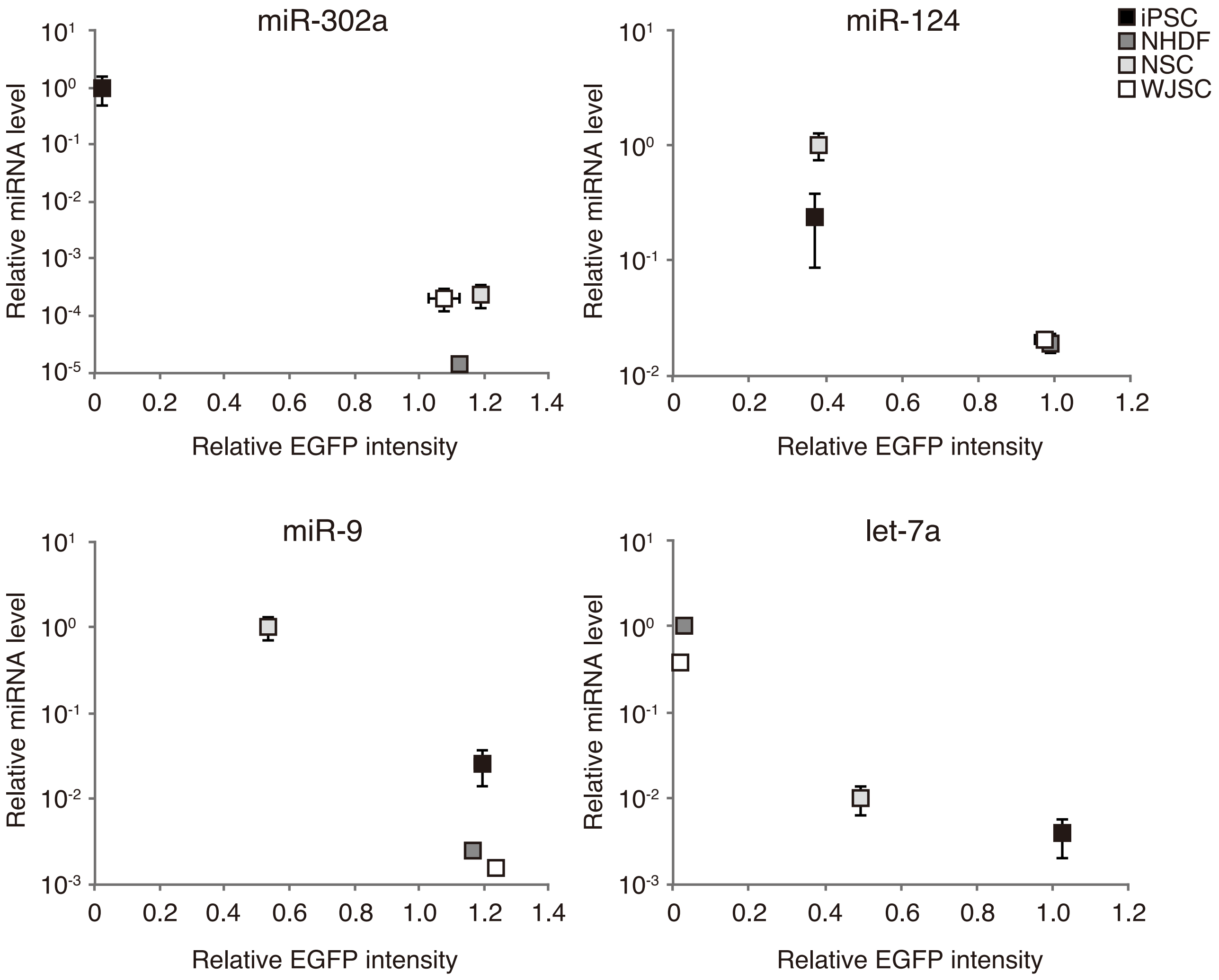


**Supplementary Figure S4. Correlation between EGFP suppression and miRNA levels.** Relative EGFP intensity of each cell infected with SeVdp-miR-Sensor compared to that of SeVdp-FlucT-infected cells (shown in Fig. 1e,f, and Supplementary Fig. S3c,d) and relative miRNA levels in each cell (shown in Supplementary Fig. S2a,b,c) were plotted to determine their relationship. Values are the same as those described for the original figures.

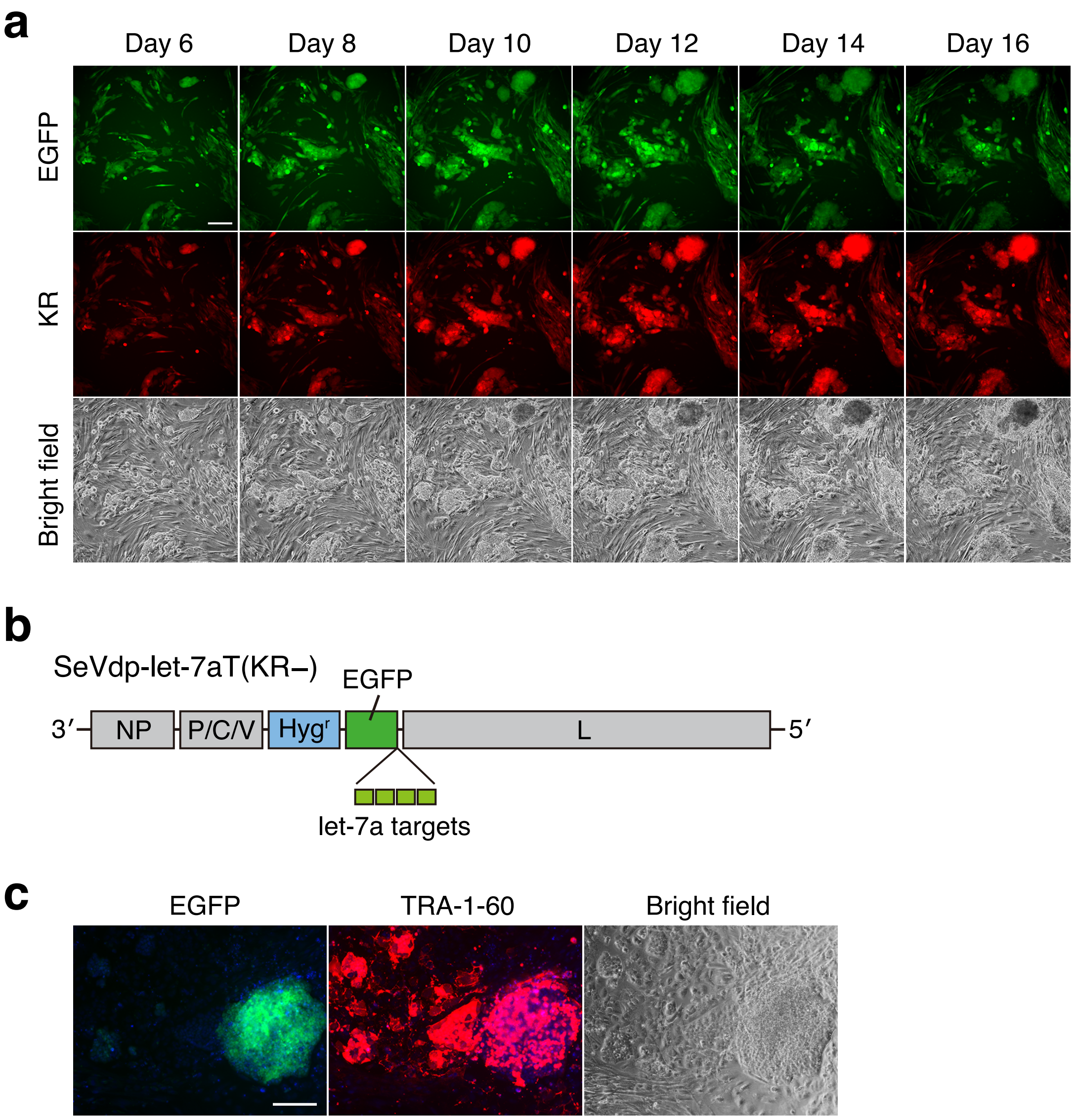


**Supplementary Figure S5. Fluorescence imaging analysis of hiPSC generation.** (a) Time-lapse imaging analysis during hiPSC generation. NHDFs were co-infected with SeVdp(KOSM) and SeVdp-FlucT, and EGFP and KR expression was examined. Scale bar: 200 μm. (b) Structure of SeVdp-let-7aT(KR–). Four copies of the complementary sequence for let-7a were incorporated into the 3 ′ UTR of *EGFP*. (c) TRA-1-60 and let-7 expression in reprogrammed colonies. EGFP and TRA-1-60 expression was examined on day 19. Scale bar: 200 μm.

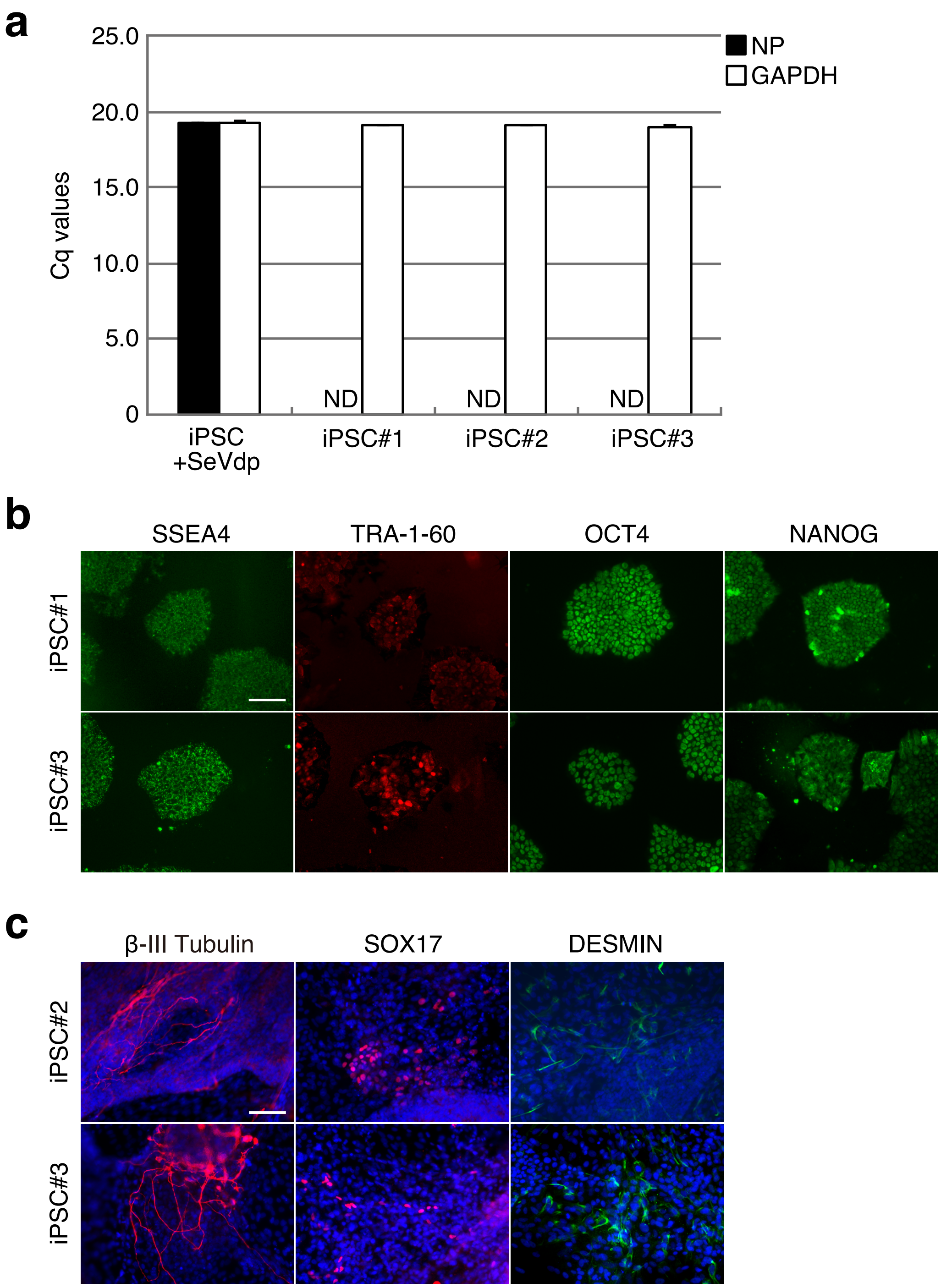


**Supplementary Figure S6. Characterization of hiPSCs.** (a) Expression of the SeV *NP* gene in hiPSC clones. The *NP* and *GAPDH* mRNA levels were determined by qRT-PCR analysis, and Cq (quantification cycle) values are shown. hiPSCs infected with SeVdp-FlucT was used as a positive control. ND indicates not detected. The mean and standard deviation (n = 3) are presented. (b) Expression of ESC markers in hiPSCs. The expression of SSEA4, TRA-1-60, OCT4, and NANOG in hiPSC clones (#1 and #3) was analysed by immunofluorescence staining. Scale bar: 200 µm. (c) Differentiation of hiPSCs. hiPSC clones (#2 and #3) were differentiated into three germ layers: ectoderm (β-III Tubulin), endoderm (SOX17), and mesoderm (DESMIN). Scale bar: 100 µm.

**Supplementary Table S1. The complementary sequences of target miRNAs.**

| Target miRNA | Sequence (5′ to 3′) |
|---|---|
| let-7a | AACTATACAACCTACTACCTCACAGAATAACTATACAACCTACTACCTCAATCAGAAACTATACAACCTACTACCTCATCCATTAACTATACAACCTACTACCTCA |
| miR-9 | TCATACAGCTAGATAACCAAAGACAGAATTCATACAGCTAGATAACCAAAGAATCAGATCATACAGCTAGATAACCAAAGATCCATTTCATACAGCTAGATAACCAAAGA |
| miR-17 | CTACCTGCACTGTAAGCACTTTGCAGAATCTACCTGCACTGTAAGCACTTTGATCAGACTACCTGCACTGTAAGCACTTTGTCCATTCTACCTGCACTGTAAGCACTTTG |
| miR-124 | GGCATTCACCGCGTGCCTTACAGAATGGCATTCACCGCGTGCCTTAATCAGAGGCATTCACCGCGTGCCTTATCCATTGGCATTCACCGCGTGCCTTA |
| miR-302a | TCACCAAAACATGGAAGCACTTACAGAATTCACCAAAACATGGAAGCACTTAATCAGATCACCAAAACATGGAAGCACTTATCCATTTCACCAAAACATGGAAGCACTTA |
| firefly luciferase (21 nucleotides) | CTTACGCTGAGTACTTCGAAACAGAATCTTACGCTGAGTACTTCGAAAATCAGACTTACGCTGAGTACTTCGAAATCCATTCTTACGCTGAGTACTTCGAAA |
| Scramble | GTGCTCTAACCTTCTCGTAAGACAGAATGTGCTCTAACCTTCTCGTAAGAATCAGAGTGCTCTAACCTTCTCGTAAGATCCATTGTGCTCTAACCTTCTCGTAAGA |

## Supplementary Video legends

**Supplementary Video S1. Calcium imaging of converted neuronal cells.** MEFs were infected with SeVdp(ABMN), and calcium activity was measured at 12 days post-infection.

**Supplementary Video S2. Time-lapse imaging of miR-124 expression during direct neuronal conversion.** MEFs were co-infected with SeVdp(ABMN) and SeVdp-124T, and (a) EGFP and (b) KR expression was examined by time-lapse fluorescence microscopy. As a control experiment, SeVdp-scrT was used instead of SeVdp-124T, and (c) EGFP and (d) KR expression was examined as described for (a) and (b).

# Full-length images of gels presented in Figure 3c

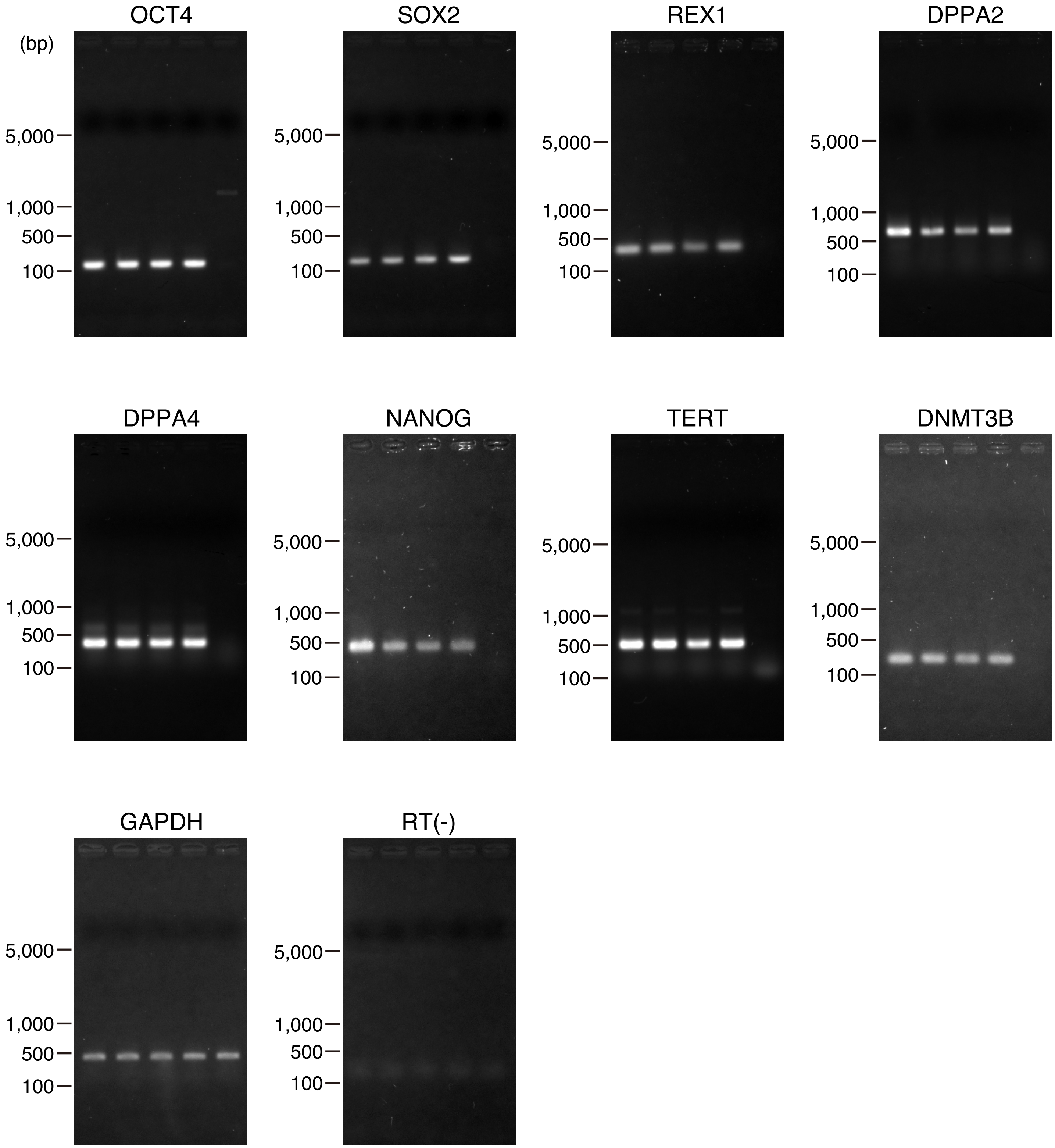

Supplement: Supplementary file 1 — Supplementary Information [file 41598_2017_12847_MOESM1_ESM.pdf]
